# Dendritic cells under allergic condition enhance the activation of pruritogen-responsive neurons via inducing itch receptors in a co-culture study

**DOI:** 10.1186/s12865-024-00604-4

**Published:** 2024-02-12

**Authors:** Tichakorn Singto, Viviane Filor, Jonathan Vidak, Robert Klopfleisch, Wolfgang Bäumer

**Affiliations:** 1 Institute of Pharmacology and Toxicology, Department of Veterinary Medicine, Freie Universität Berlin, Koserstraße. 20, Berlin, 14195 Germany; 2 Institute of Animal Pathology, Department of Veterinary Medicine, Freie Universität Berlin, Robert-von- Ostertag-Straße 15, Berlin, 14163 Germany

**Keywords:** Dendritic cell, Dorsal root ganglia, Peripheral sensitization, Itch, Pruritus, Allergic contact dermatitis

## Abstract

**Background:**

Itch sensitization has been reported in patients with chronic allergic skin diseases and observed in a mouse model of allergic contact dermatitis (ACD). There is evidence suggesting that neuroimmune interactions may contribute to itch sensitization, as an increase in dendritic cells (DCs) within ganglia has been observed during allergic conditions. However, how DCs interact with sensory neurons in ganglia during allergic conditions is still not known. This study aims to investigate the role of DCs in dorsal root ganglion (DRG) under ACD conditions, specifically focusing on itch sensitization within the DRG. The tolylene-2,4-diisocyanate (TDI) mouse model for ACD and the co-culture model of DCs and DRG neurons was employed in this study.

**Results:**

We successfully induced ACD by TDI, as evidenced by the development of edema, elevated total serum IgE levels, and an observed itch reaction in TDI-sensitized mice. Calcium imaging and RT-qPCR analysis revealed that TDI-sensitized mice exhibited signs of peripheral sensitization, including a higher percentage of neurons responding to pruritogens and increased activation and expression of itch receptors in excised DRG of TDI-sensitized mice. Immunofluorescence and flow cytometric analysis displayed an increase of MHCII^+^ cells, which serves as a marker for DCs, within DRG during ACD. The co-culture study revealed that when DRG neurons were cultured with DCs, there was an increase in the number of neurons responsive to pruritogens and activation of itch receptors such as TRPA1, TRPV1, H1R, and TRPV4. In addition, the immunofluorescence and RT-qPCR study confirmed an upregulation of TRPV4.

**Conclusions:**

Our findings indicate that there is an increase of MHCII^+^ cells and itch peripheral sensitization in DRG under TDI-induced ACD condition. It has been found that MHCII^+^ cells in DRG might contribute to the itch peripheral sensitization by activating itch receptors, as shown through co-culture studies between DRG neurons and DCs. Further studies are required to identify the specific mediator(s) responsible for peripheral sensitization induced by activated DCs.

**Supplementary Information:**

The online version contains supplementary material available at 10.1186/s12865-024-00604-4.

## Background

Allergic contact dermatitis (ACD) is one of the most common occupational skin diseases [1, 2]. It is an immune reaction of delayed-type IV sensitivity, presenting with symptoms such as edema, erythema, pruritus, scaling and vesicles on the skin [3]. Especially, pruritus, also known as itch, is a main symptom of ACD. Not only does itch significantly impact quality of life but it also exacerbates the progression of the disease. The itch transmission pathway begins in the skin where activated immune cells and skin cells are present. Upon activation, these cells release itch mediators e.g., histamine, serotonin, tryptase, endothelin-1, nerve growth factor, interleukin 4 (IL-4), interleukin 13 (IL-13), interleukin 31 (IL-31), interleukin 33, sphingosine-1-phosphate, neurotrophins, substance P and thymic stromal lymphopoietin. These mediators can trigger specific receptors on unmyelinated C afferent nerve fibers and A-delta fibers in the skin, with the cell bodies being in dorsal root ganglia (DRG) [3–5]. Several itch receptors are G-protein-coupled receptors (GPCRs) like the histamine receptors 1–4 (H1R-H4R), Mas-related G protein-coupled receptors (Mrgprs). The downstream signal transduction pathways via these GPCRs mainly require activation of transient receptor potential channel vanilloid 1 (TRPV1) and transient receptor potential channel ankyrin 1 (TRPA1) [6, 7]. The itch primary sensory neurons make synaptic connections with interneurons in the spinal cord and finally transmit itch stimuli to the brain [6, 8, 9].

Peripheral sensitization is defined as an increased sensitivity of peripheral afferent nerves to pruritogens, manifested by a reduction in the threshold and an increase in the magnitude of the response. Itch sensitization has been reported to occur in patients with chronic allergic skin diseases [10]. Chronic pruritic animals also display signs of peripheral sensitization with an increase in pruritogen-responsive neurons observed in DRG [11]. It was indicated that a neuroimmune interaction might play a role in peripheral sensitization [12]. Activated immune cells that locate close to sensory nerve fibers activate itch sensory neurons by releasing inflammatory mediators leading to itch transmission and sensitizing sensory neurons. In turn, sensory nerve endings release neuromodulators into the skin which have an impact on immune cells, thereby contributing to inflammation, barrier dysfunction, and itch [13].

Neuroimmune interaction was observed in a mouse model of allergic airway inflammation, which showed an increase in the number of DCs migrating into airway sensory ganglia [14]. Also, an increase of DCs was found in DRG under tolylene-2,4-diisocyanate (TDI)-induced allergic chronic pruritus in a mouse model [11]. However, how DCs interact with sensory neurons in ganglia during allergic conditions is still not known. Expanding our knowledge of the peripheral sensitization and the role of DCs in DRG will enhance our understanding of the pathophysiology of itch. This could have potential benefits for itch treatment, as no single drug can provide complete effectiveness. Therefore, in this study we investigated the contribution of major histocompatibility complex class II positive cells (MHCII^+^ cells) in DRG under allergic condition using the TDI-induced ACD mouse model and a co-culture model of DCs and DRG neurons.

## Methods

### TDI-induced chronic allergic contact dermatitis mouse model

**Fig. 1:**
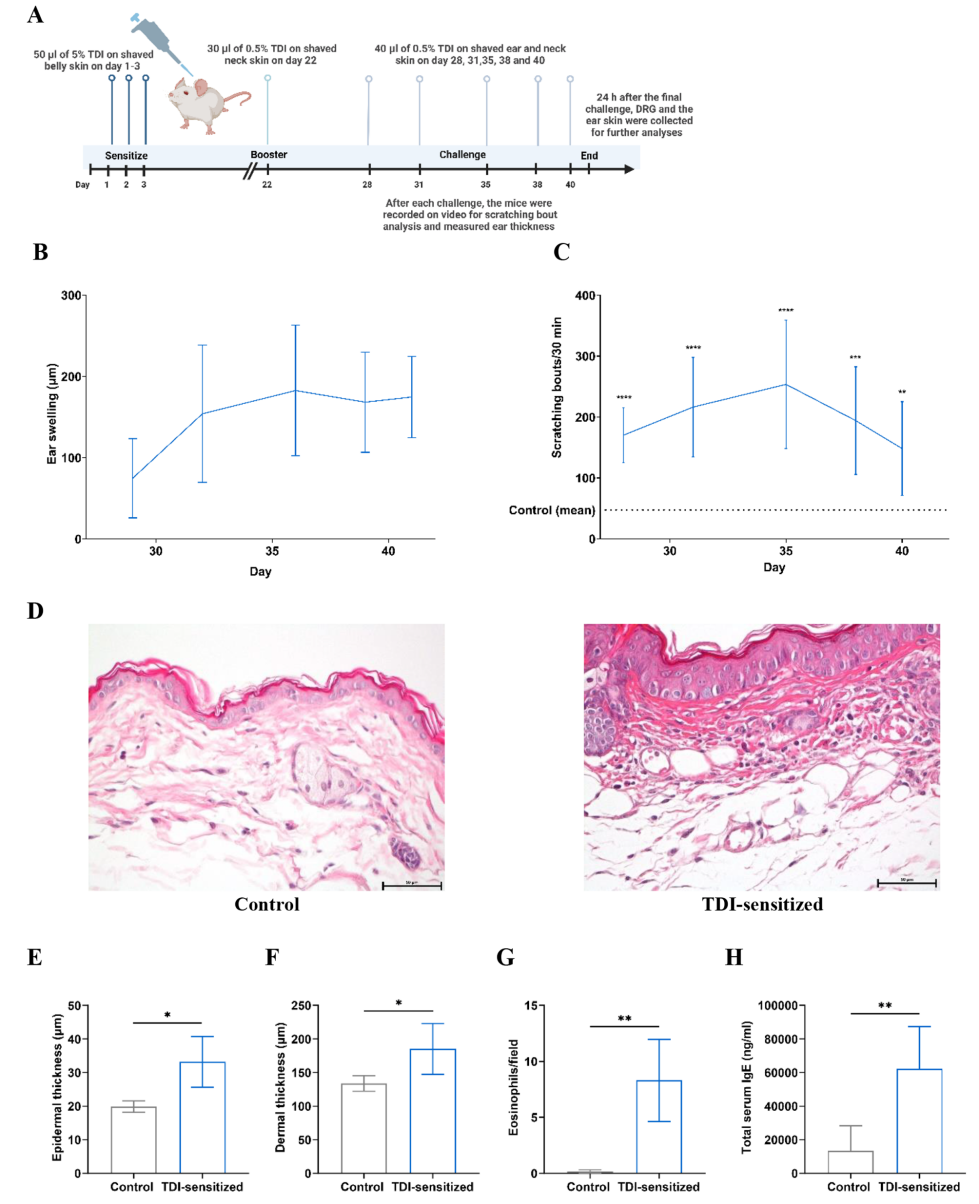
TDI-induced chronic ACD mouse model. (**A**) Protocol for TDI-induced chronic ACD mouse model. (**B**) Ear swelling of TDI-sensitized group during the challenge phase of TDI-induced ACD. (**C**) Itch reaction during the challenge phase of TDI-induced ACD. Scratching bouts were monitored from day 28 to day 40. (**D**) Representative images of H&E staining of ear skin of control and TDI-sensitized group. Summary of epidermal (**E**) and dermal (**F**) thickness of ear skin. (**G**) Summary of the number of eosinophils per observation field. (**H**) The total serum IgE levels. Results are expressed as mean ± S.D. (*N* = 8 * *p* < 0.05, ** *p* < 0.01, *** *p* < 0.001 and **** *p* < 0.01, Independent t-test vs. control group)

BALB/c mice (8–12 weeks old, 8 males and 8 females) were bred under a protocol approved by the local animal welfare committee (Reg-Nr: 0234/17). The mice were then randomly assigned to the control and TDI-sensitized group, 4 males and 4 females (*N* = 8) per group. Housing facilities were maintained at 23 ± 2 °C and 36–57% relative humidity, and a 12-h light–dark cycle. Chronic ACD was induced by sensitization and repetitive challenge with TDI (Sigma-Aldrich, Taufkirchen, Germany, T39853) following a previous study of our research group [11]. One day before the experiment began, the mice were prepared by removing their abdominal hair using electric clippers, followed by depilatory cream, and then tape stripped in that area 10 times. On day 1, 50 µl of 5% TDI in acetone was applied topically on their belly. On day 2 and 3, 20 µl of 5% TDI was also applied to their belly. On day 22, 30 µl of 0.5% TDI was applied to shaved neck. For the challenge phase, 40 µl of 0.5% TDI was applied to the shaved neck and left ear skin five times on day 28, 31, 35, 38 and 40. After each challenge, the mice were placed in observation chambers and recorded on video for scratching bout analysis and measured ear thickness by a caliper. The scratching bouts consist of repetitive scratching movements by a hind paw in the TDI-applied areas until the paw is licked by the mouse or placed on the ground [15]. Ear swelling was calculated by a comparison of the ear thickness before and after each challenge time for 24 h. Twenty-four hours after the final challenge, the mice were euthanized via CO_2_ asphyxiation and their DRG, blood and the ear skin were collected to conduct additional analyses (Fig. 1A). The blood was collected and investigated total serum Immunoglobulin E (IgE) using a mouse enzyme-linked Immunosorbent Assay (ELISA) kit (Invitrogen, Massachusetts, USA).

### Histological evaluation of ear skin

The ear skin was fixed in 10% formaldehyde and embedded in paraffin. Skin sections (5 μm) were stained with hematoxylin and eosin (H&E) following standard protocol. Epidermal and dermal layer thickness and eosinophil infiltration were evaluated using Nikon Eclipse Ni-E microscope (Amstelveen, Netherlands) and NIS-Elements software with a 20X objective lens. To assess the thickness, 20 measurements per mouse were combined to obtain microscopic data on thickness while 10 fields were evaluated per mouse to determine the number of eosinophils present in each field.

### Isolation of DRG neurons

After the mice were euthanized, the spinal column was opened. Ten to twelve DRG were excised from the vertebral level where TDI was applied and enzymatically digested in 2.5 mg/ml dispase II (Sigma-Aldrich, Taufkirchen, Germany) and 2.5 mg/ml collagenase type I (Alfa Aesar, MA, USA) in Ca^2+^ and Mg^2+−^free Hank’s Buffered Salt Solution (Coutiva, Utah, USA) for 60 min at 37 ºC. The cell solution was dissociated by trituration using Pasteur pipettes. Cells were washed in DRG medium (Dulbecco’s modified Eagle medium, Corning, VA, USA) with 10% fetal bovine serum (FBS, Biowest, Nuaillé, France) and Pen-strep solution (100 U/mL streptomycin and 100 U/mL penicillin (Corning, VA, USA)) by centrifugation for three times. The cell pellet was resuspended in 120 µl DRG medium and placed onto 0.1 mg/ml poly-L-lysine (Sigma-Aldrich, Taufkirchen, Germany) and 5 µg/ml laminin (Roche, Mannheim, Germany)-coated coverslips (20 µl cell suspension per a coverslip). The DRG neurons were incubated at 37 °C for 2 h and then flooded with an additional 1 ml DRG medium and further incubated at 37 °C for 16 h before performing calcium imaging or co-culture study [11].

### Calcium imaging

Cultured DRG neurons were loaded with 2 µg/ml Fura-2 AM (Sigma-Aldrich, Taufkirchen, Germany) and incubated at 37 °C 5% CO_2_ protected from light for 30 min. Fluorescence at 340 and 380 nm excitation (510 nm emission) wavelengths were recorded on an inverted microscope (Leica Dmi8, Wetzlar, Germany). Fluorescence at 340 and 380 nm excitation wavelengths were recorded every hundred milliseconds through the use of a camera connected to a Lambda LS lamp and an optical filter changer from Lambda, with software calculation of the Fura-2 AM ratio (F340/F380). The F340/F380 ratio rises to indicate an increase in intracellular Ca^2+^ concentration, indicating that the sensory neuron has been activated. Responses were obtained from 30 to 70 neurons on each coverslip. During the imaging process, a consistent flow of buffer solution (pH 7.4; 136 mM NaCl, 5.4 mM KCl 2.9 mM CaCl_2_, 2.5 mM MgCl_2_, 10.9 mM D-glucose, 0.6 mmol NaHPO_4_, and 14.3 mmol/l NaHCO_3_) was maintained at a rate of 3 ml/min, 37 °C and a pH level of 7.3–7.4. One millimolar histamine (Sigma-Aldrich), 10 µM allyl isothiocyanate (AITC) (Sigma-Aldrich,), 1 µM capsaicin (Sigma-Aldrich) and 1.5 µg/ml compound 48/80 (Sigma-Aldrich), 100 µM histamine trifluromethyl toluidine (HTMT) (Sigma-Aldrich), 0.1 mM ST-1006 (provided by Prof. Dr. H. Stark (Heinrich-Heine‐Universität, Düsseldorf, Germany)), 5 nM GSK1016790A (Sigma-Aldrich) and 0.1 mM Amthamine (Tocris, Bristol, UK) were applied. Neuronal viability was obtained through the application of 150 mM KCl, which resulted in an increase in Fura-2 ratio (F340/F380). Activation of neurons was considered when there was an elevation by more than 10% from the initial resting level subsequent to chemical application. The analysis only included viable cells that responded to KCl [11, 16].

### Immunofluorescence

The immunofluorescence procedure was modified from the previous study [14]. Five to seven DRG from each mouse were fixed in Zamboni solution (Morphisto, Frankfurt am Main, Germany) for 6 h at 4 °C. The fixed DRG were incubated in 0.1 M phosphate buffered saline (PBS, Thermo Fisher Scientific, MA, USA) overnight and followed with 30% sucrose in 0.1 M PBS at 4 °C for 24 h. The fixed DRG were embedded in tissue freezing medium (Leica, Wetzlar, Germany) and frozen in a -80 °C freezer. Ten µm sections of DRG were prepared using a microtome cryostat (Microm Cryo-Star HM 560, Walldorf, Germany), placed on THERMO Superfrost slides (Thermo Fisher Scientific, MA, USA), dried at room temperature for 1 h and subsequently stored in the − 80 °C freezer. Three slides with 12 sections per slide of each mouse were rehydrated with PBS solution for 5 min at room temperature. The sections were incubated with 0.5% triton X-100 (Sigma-Aldrich) in PBS for 10 min followed by blocking solution (5% donkey serum and 0.1% triton X-100 in PBS) for 30 min at room temperature. Primary antibodies (listed in Table 1) were applied to the sections overnight at 4 °C. Subsequently, the sections were incubated for two hours at room temperature with secondary antibodies conjugated with fluorescein (listed in Table 1). The sections were washed twice with diluted 1:10 PBS, once with distilled water, and mounted on fluorescent mounting medium containing DAPI for counterstaining (Invitrogen, MA, USA). A quantitative analysis of positive cells was carried out using a fluorescence microscope (Leica Dmi8) and ImageJ (National Institutes of Health, USA) software for cell counting.

**Table 1:** List of antibodies used in this study

Antibody	Source	Dilution
**Primary antibodies**		
Mouse anti-NeuN antibody	Merck, Darmstadt, Germany	1:500
Rabbit anti-TRPV4 antibody	Abcam, Cambridge, UK	1:400
Rabbit anti-CD68 antibody	Abcam, Cambridge, UK	1:200
Rabbit anti-arginase antibody	Abcam, Cambridge, UK	1:200
Rat anti-CD19 antibody	BioLegend, CA, USA	1:200
Rat anti-IA/IE antibody	BioLegend, CA, USA	1:200
**Secondary antibodies**		
Alexa Fluor 488-cojugated donkey anti-rabbit IgG	Jackson ImmunoResearch, Baltimore, USA	1:400
Cy3-conjugated donkey anti-rat IgG		
Alexa Fluor 488-cojugated donkey anti-mouse IgG		
Alexa Fluor 647-cojugated donkey anti-rabbit IgG		

### Reverse transcription-quantitative polymerase chain reaction

The expression level of specific genes was measured using reverse transcription-quantitative polymerase chain reaction (RT-qPCR). Before RT-qPCR analysis total RNA was extracted by RNAeasy Mini Kit (Qiagen, Venlo, Netherland) and reverse transcribed into cDNA by QuantiTech QuantiTect Reverse Transcription Kit (Qiagen). Primers were designed by PrimerBank [17] and provided by Sigma-Aldrich (MA, USA) (Table 2). To ensure accurate quantification of gene expression levels, the obtained value was normalized with Glyceraldehyde-3-phosphate dehydrogenase (*GAPDH*) and Ribosomal protein L13a (*RPL13A*). Real-time detection of the amount of double-strand DNA synthesized during PCR was achieved using SYBR Green master mix (Qiagen). The delta-delta Ct method by Bio-Rad CFX Maestro version 2.3 was utilized to calculate gene expression fold change relative to a control group. RT-qPCR products were validated by agarose gel electrophoresis.

**Table 2:** List of primers used in this study

Gene	Forward primer (5’>3’)	Reverse primer (5’>3’)	Accession number
*IL31RA*	TCCTGATGTTCCCAACCCTG	TTAGGACCACGTCTTCTGTGT	NM_139299
*OSMR*	CATCCCGAAGCGAAGTCTTGG	GGCTGGGACAGTCCATTCTAAA	NM_011019
*TRPV4*	ATGGCAGATCCTGGTGATGG	GGAACTTCATACGCAGGTTTGG	NM_022017
*H1R*	CAAGATGTGTGAGGGGAACAG	CTACCGACAGGCTGACAATGT	NM_008285
*H2R*	CCCAATGGCACGGTTCATTC	GCCGACGATTCAAGCTGACA	NM_001010973.2
*H3R*	CTCCGCACCCAGAACAACTT	GCACGATGTTGAAGACTGAGG	NM_133849.3
*MRGPRA3*	CTCAAGTTTACCCTACCCAAAGG	CCGCAGAAATAACCATCCAGAA	NP_694707
*MRGPRC11*	TCTCATCCCACGACACAGAAT	AGCCAGAGTACAATGGTGTTTC	NM_207540
*MRGPRD*	TTTTCAGTGACATTCCTCGCC	GCACATAGACACAGAAGGGAGA	NM_203490
*PAR2*	TGCTGGGAGGTATCACCCTTC	GCTGGGTTTCTAATCTGCCAAT	NM_007974
*TRPA1*	GTCCAGGGCGTTGTCTATCG	CGTGATGCAGAGGACAGAGAT	NM_177781
*TRPV1*	CCGGCTTTTTGGGAAGGGT	GAGACAGGTAGGTCCATCCAC	NM_001001445.2
*GAPDH*	TGACCTCAACTACATGGTCTACA	CTTCCCATTCTCGGCCTTG	XM_036165840.1
*RPL13A*	AGCCTACCAGAAAGTTTGCTTAC	GCTTCTTCTTCCGATAGTGCATC	NM_009438.5

### Bone marrow derived dendritic cells -DRG neurons co-culture study

Bone marrow derived dendritic cells (BMDCs) were generated from bone marrow cells of 8–20 weeks-old BALB/c wild-type mice following previously described methods [18]. The bone marrow cells were cultured in RPMI-1640 supplemented with 100 U/ml penicillin, 100 U/ml streptomycin, 50 mM 2-mercaptoethanol, 10% heat-inactivated FBS, and 10–20 ng/ml granulocyte–macrophage colony-stimulating factor (GM-CSF)). The bone marrow cells were cultured in 5% CO_2_ at 37 °C for 10 days until they differentiated into BMDCs. The purity of BMDC culture was measured for surface expression of MHCII and CD11c, the classic DC markers, by flow cytometry. DRG neurons were isolated using a similar procedure as described above, which involves preparing DRG for calcium imaging analysis. In the co-culture study, 1.5 × 10^5^ BMDCs were added to DRG cultures that had been prepared on a coverslip earlier for 16 h. A co-culture medium was prepared by mixing 50% DC medium and 50% DRG medium. The co-cultures were stimulated by incubating with 20 ng/ml IL-4 (Peprotech, Hamburg, Germany), 20 ng/ml IL-13 (Peprotech, Hamburg, Germany) and 10 ng/ml lipopolysaccharide (LPS, Sigma-Aldrich, Taufkirchen, Germany) in 5% CO_2_ at 37 °C for 16 h. The supernatant of the co-culture was collected and investigated for tumor necrosis factor alpha (TNF-α) and Interleukin 1 beta (IL-1β) secretion using a mouse ELISA kit (R&D Systems, Boston, USA). The co-culture system’s coverslip component was used to investigate the response of DRG to substances related to itching by calcium imaging. Additionally, the expression of the transient receptor potential channel vanilloid 4 (TRPV4) in the DRG neurons was examined using RT-qPCR and immunofluorescence. For indirect coculture, a transwell insert (Greiner bio-one, Frickenhausen, Germany) with a pore size of 0.4 μm was used to separate direct cell-cell contact. DRG neurons were cultured in the lower chamber, while BMDCs were added in the insert.

### Language and grammar correction

The manuscript was checked and corrected by a large language model via https://jenni.ai/.

### Statistical analysis

All data were statistically analyzed using GraphPad Prism 9.5.1 software (CA, USA). Independent samples t-test was applied to the obtained data. Where required, Fisher’s exact test was utilized. At least three independent observations were conducted, and data are presented as individual values together with mean ± standard deviation (SD). *P* < 0.05 or 0.01 was considered statistically significant.

## Results

### TDI induced allergic contact dermatitis and itch reaction in mice

After each challenge with TDI, scratching bouts and ear thickness were analyzed. Distinct ear swelling was observed in the TDI-sensitized group (*N* = 8, Fig. 1B). The TDI-sensitized group showed an increase in scratching bouts compared to the control group (*N* = 8, Fig. 1C). The ear swelling and scratching bouts increased continuously over the third challenge of TDI and reached a plateau or decreased slightly by the fourth challenge. Histological analysis of the ear skin and serum total IgE levels were investigated 24 h after the last challenge. A significant increase in the thickness of both the epidermis (Control 19.84 ± 1.69 μm vs. TDI-sensitized 33.22 ± 7.55 μm) and dermis (Control 133.87 ± 11.63 μm vs. TDI-sensitized 185.09 ± 37.99 μm) was noted in the TDI-sensitized group (*p* < 0.05, *N* = 8, Fig. 1D and F). The TDI-sensitized group showed also a higher number of eosinophils per field in the ear skin compared to control (Control 0.15 ± 0.17 cells/field vs. TDI-sensitized 8.30 ± 3.68 cells/field, *p* < 0.01, *N* = 8, Fig. 1G). As shown in Fig. 1H, the TDI-sensitized group exhibited a significant increase in total serum IgE levels compared to the control group, with a five-fold difference (Control 13,438 ± 14,929 ng/ml vs. TDI-sensitized 62,378 ± 25,000 ng/ml, *p* < 0.01, *N* = 8). Overall, the mice that were treated with TDI displayed typical signs of ACD.

### Signs of peripheral sensitization was observed in TDI-sensitized mice

**Fig. 2:**
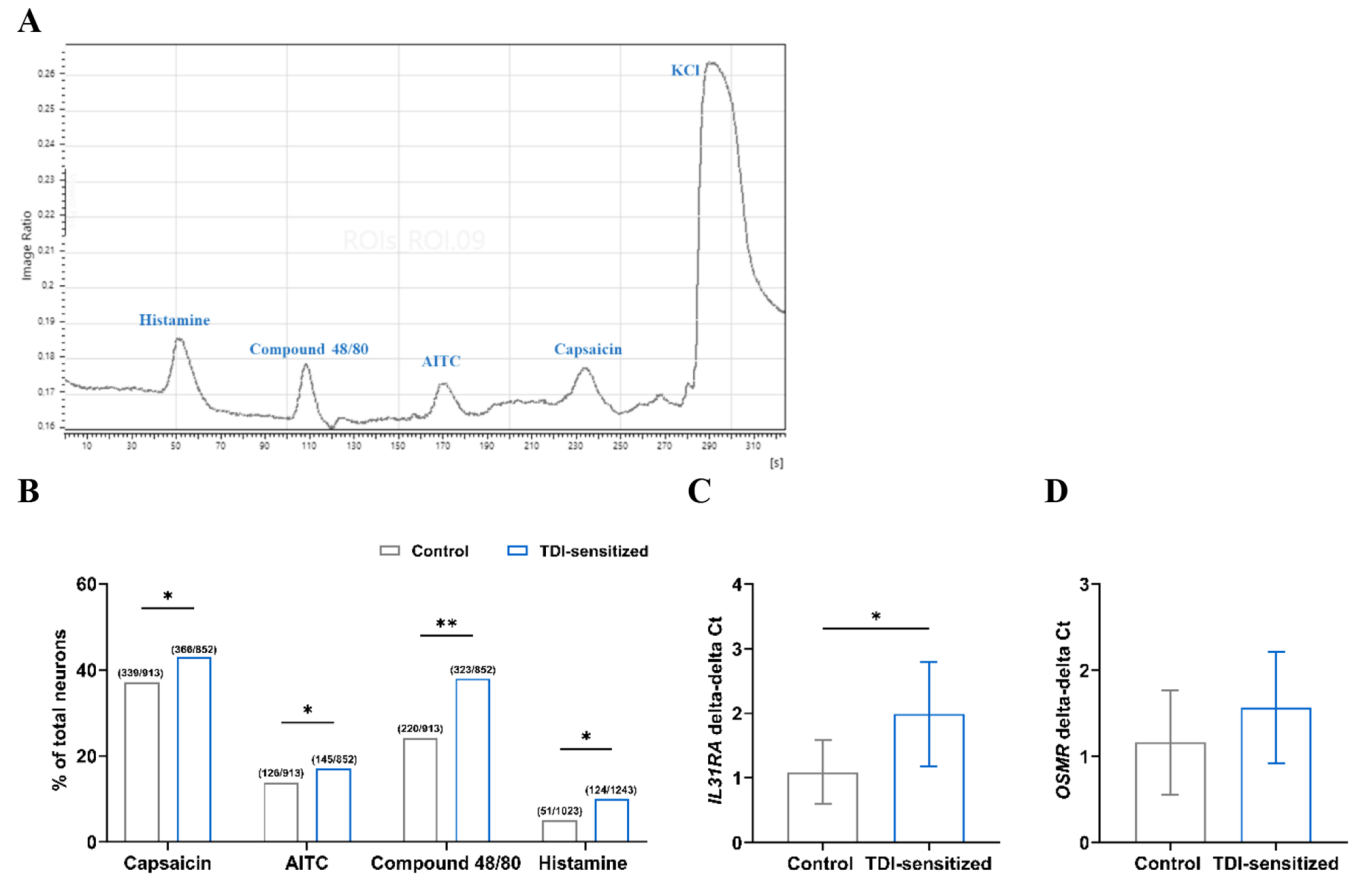
Signs of peripheral sensitization in TDI-sensitized mice. (**A**) Representative tracing of Ca^2+^ response (increased F340/F380) for a single neuron to pruritogens and agonists of itch receptors. (**B**) Substances induced intracellular Ca^2+^ increase in mouse sensory neuron showed a significant response to capsaicin, AITC, compound 48/80 and histamine in DRG neurons of TDI-sensitized group. Data are presented as the percentage of responsive cell per total DRG neuron (*N* = 8, * *p* < 0.05, ** *p* < 0.01, Fisher exact test). The relation of expression of *IL31RA* (**C**) and *OSMR* (**D**) mRNA in DRG of TDI-sensitized group comparing to control. Results are expressed as mean ± S.D. (*N* = 3, * *p* < 0.01, Independent t-test vs. control group)

The peripheral sensitization was investigated by assessing the responsiveness to pruritogens, as well as the activation and expression of receptors associated with itch in excised DRG neurons. The pruritogen-responsive neurons were analyzed by calcium imaging comparing the TDI-sensitized group with a control group. It was found that there was a higher percentage of neurons responding to histamine (control 4.99% vs. TDI-sensitized 9.98%, *p* < 0.05) and compound 48/80 (control 24.10% vs. TDI-sensitized 37.91%, *p* < 0.01) in the TDI-sensitized group compared to the control group (*N* = 8, Fig. 2A and B). The neurons responsive to an agonist of each receptor were measured to investigate the activation of itch-related receptors. DRG neurons excised from TDI-sensitized mice had a higher percentage of neurons responding to agonists of TRPA1 (AITC: control 13.08% vs. TDI-sensitized 17.02%, *p* < 0.05) and TRPV1 (capsaicin: control 37.13% vs. TDI-sensitized 42.96%, *p* < 0.05) compared to control (*N* = 8, Fig. 2A and B). The gene expression of IL-31 receptor A (*IL31RA*) and oncostatin M receptor (*OSMR*), which build the receptor for IL-31, was investigated using RT-qPCR. The DRG neurons excised from the TDI-sensitized mice displayed an upregulation of *IL31RA* by 2 times compared to the control group (*p* < 0.01, *N* = 3, Fig. 2C and Figure A1.1 in an additional file 1). *OSMR* was upregulated in the TDI-sensitized group by 1.9 times compared to the control group but did not reach a significant difference (*p* > 0.05, *N* = 3, Fig. 2D and Figure A1.1 in the additional file 1).

**Fig. 3:**
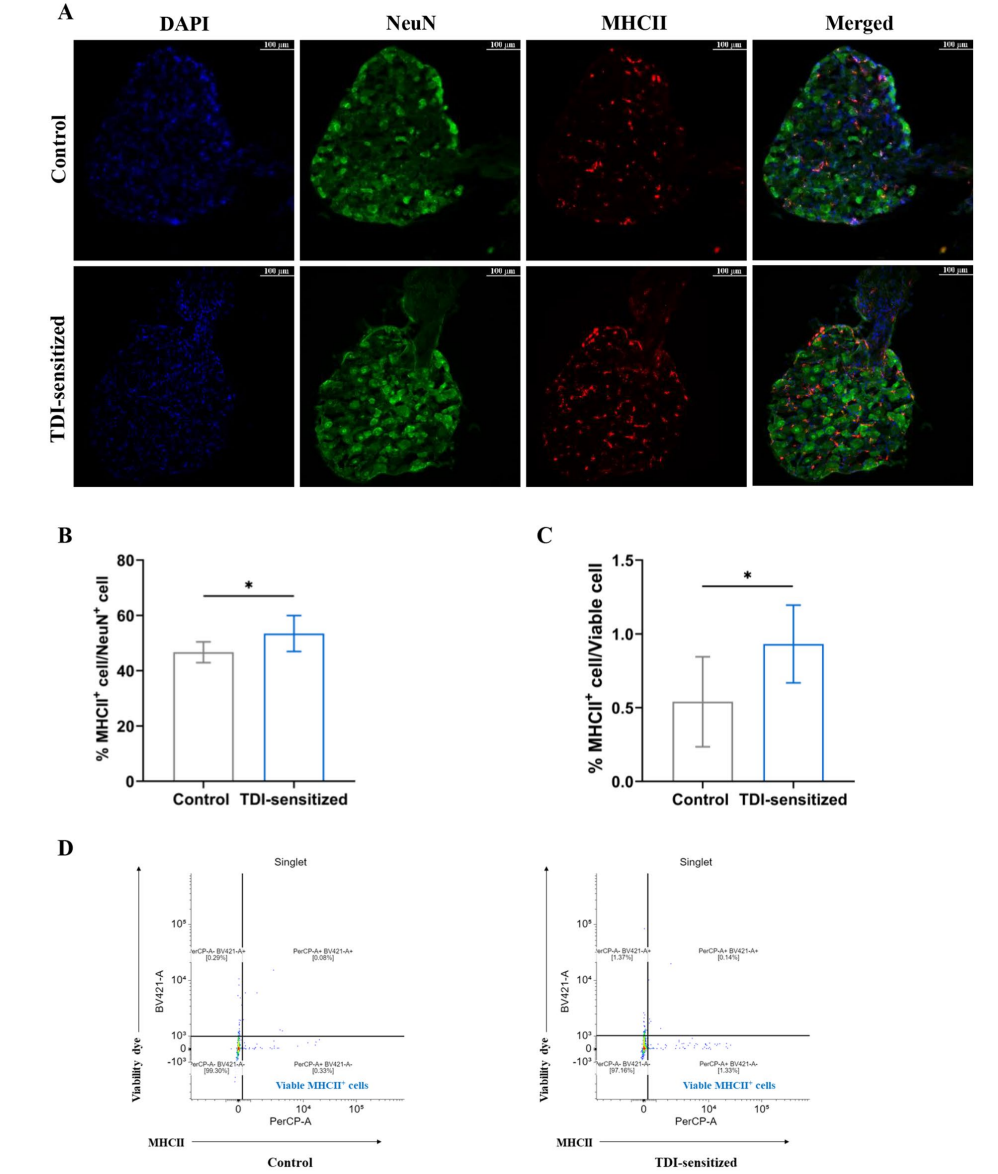
An increase of MHCII^+^ cells in DRG under allergic contact dermatitis condition. (**A**) Zamboni fixed sections of DRG were stained with MHC II for DCs and NeuN for neurons. The images were taken with a fluorescence microscope (Leica Dmi8, Wetzlar, Germany) using a 20X objective. Immunofluorescence analysis revealed amount of MHCII^+^ cells, which are located next to the neuron in DRG of the TDI-sensitized mice as well as control and their number increased significantly during TDI-induced ACD. (**B**) The quantification of MHCII^+^ cells in DRG of control and TDI-sensitized mice. The results were expressed as percentages of MHC^+^ cells in relation to NeuN^+^ neurons as mean ± S.D. (*N* = 8, * *p* < 0.05). (**C**) Flow cytometric analysis indicated an increase of percentage of MHCII^+^ cells in relation to total viable cells in DRG of TDI-sensitized mice comparing to control. Results are expressed as mean ± S.D. (*N* = 6, * *p* < 0.05). (**D**) Representative dot plot of DRG cell suspension from the TDI-sensitized and control group

### An increase of MHCII^+^ cells in DRG was observed under allergic contact dermatitis condition

**Fig. 4:**
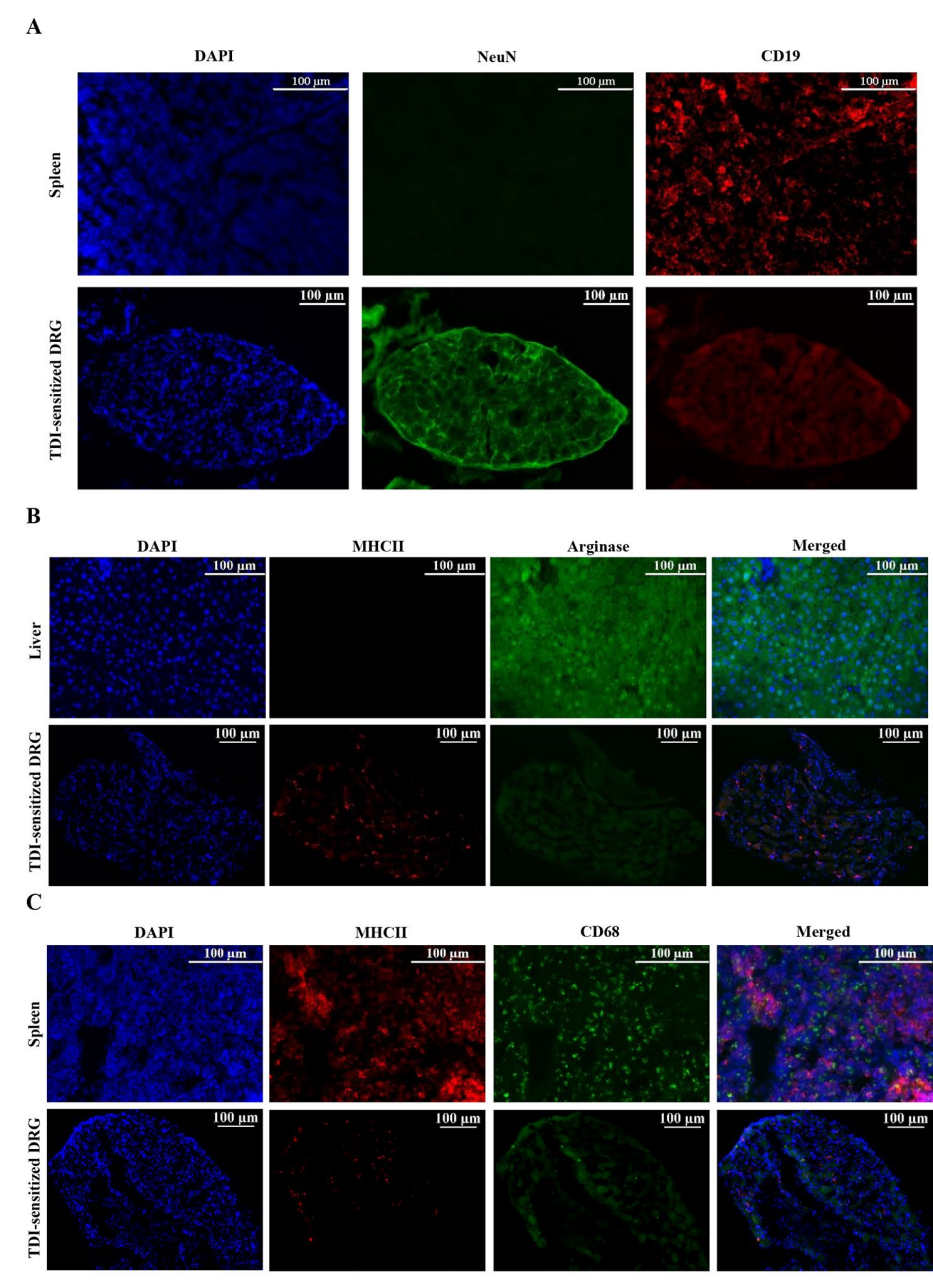
Phenotypic characteristics of MHCII^+^ cells in DRG. DRG sections were stained for different cell markers and images were acquired with fluorescence microscope (Leica Dmi8, Wetzlar, Germany) using a 20X objectives. The Immunofluorescence analysis showed the immune cells in DRG negative for CD19 (**A**) and arginase (**B**) and few MHCII^+^ cells positive for CD68 (**C**)

DRG sections were analyzed by immunofluorescence for expression of neuronal nuclear protein (NeuN) and MHCII to identify sensory neurons and MHCII^+^ cells in DRG to determine how the number of MHCII^+^ in DRG changed during ACD condition. The percentage of MHCII^+^ in relation to total neurons (NeuN^+^ cells) was significantly increased in the TDI-sensitized group (control 45.33 ± 7.02% vs. TDI-sensitized 51.32 ± 8.70%, *N* = 8, *p* < 0.05, Fig. 3A and B). Flow cytometric analysis was also conducted to examine the presence of MHCII^+^ in DRG. DCs were identified using a PerCP-conjugated MHCII antibody, which served as a marker. Figure 3C and D demonstrated that the DRG excised from the TDI-sensitized mice exhibited an increased percentage of cells expressing MHCII in relation to total viable cells compared to the control group (control 0.54 ± 0.31% vs. TDI-sensitized 0.93 ± 0.26%, *N* = 6, *p* < 0.05). In addition to the MHCII marker, additional markers including CD68, arginase and CD19 were analyzed by immunofluorescence to identify potential cell types expressing MHCII besides DCs such as macrophages and B-cells. Immune staining did not reveal any presence of CD19^+^ cell (a marker for B-cells) in the DRG (Fig. 4A). Also, the MHCII^+^ cells in the DRG did not co-express arginase (Fig. 4B). This was tested in both the TDI-sensitized group and the control group. However, approximately 8% of MHCII^+^ cells in DRG expressed CD68 (Fig. 4C).

### Activated dendritic cells increase pruritogen-responsive neurons via inducing itch receptors in an in vitro study

**Fig. 5:**
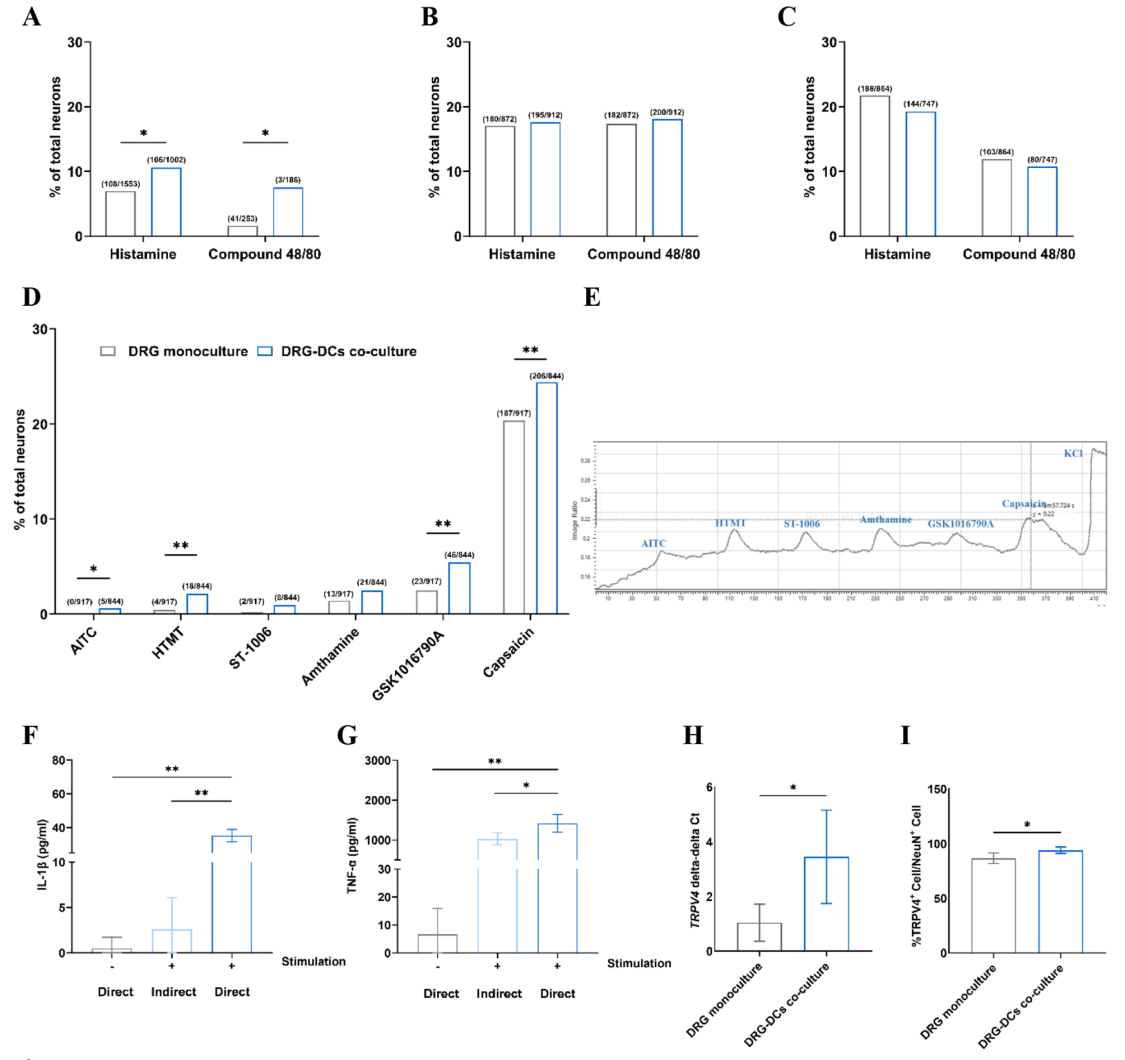
Activated dendritic cells increase pruritogen-responsive neurons via inducing itch receptors in an in vitro study. Pruritogens induced intracellular Ca^2+^ increase in mouse sensory neuron showed a significant response to compound 48/80 and histamine in DRG co-culture with DCs group comparing to DRG monoculture: (**A**) Direct co-culture with stimulation, (**B**) Indirect co-culture with stimulation and (**C**) Direct co-culture without stimulation. (**D**) Agonists of itch receptors induced intracellular Ca^2+^ increase in mouse sensory neuron showed a significant response to AITC, HTMT, GSK1016790A and capsaicin in DRG neurons of direct co-culture with DCs group. Data are presented as the percentage of responsive cell per total DRG neuron (*N* = 3, * *p* < 0.05, ** *p* < 0.01, Fisher exact test). (**E**) Representative tracing of Ca^2+^ response (increased F340/F380) for a single neuron to agonists of itch receptors. Total IL-1β (**F**) and TNF-α (**G**) Content in supernatant of co-cultures between DRG neurons and DCs. (**H**) The relation of expression of *TRPV4* in DRG of co-culture with DCs compared to DRG monoculture. (**I**) Immunofluorescence analysis of TRPV4 expression in DRG of co-culture with DCs compared to DRG monoculture. Results are expressed as mean ± S.D. (*N* = 3 * *p* < 0.05, ** *p* < 0.01, Independent t-test vs. control group)

The contribution of MHCII^+^ cells in DRG under ACD condition in the context of peripheral sensitization was investigated by the co-culture system with DRG neurons and BMDCs. The DRG neurons co-cultured with DCs were evaluated for their sensitivity to pruritogens including histamine and compound 48/80 by calcium imaging, in comparison to DRG neuron monoculture. The DRG neurons directly co-cultured with DCs exhibited a higher percentage of neurons responding to histamine and compound 48/80 (histamine: DRG monoculture 6.95% vs. DRG-DCs co-culture 10.58%; compound 48/80: DRG monoculture 1.61% vs. DRG-DCs co-culture 7.51%, *p* < 0.05, *N* = 3, Fig. 5A). To investigate whether direct cell-to-cell contact is required for the sensitization effect, an indirect co-culture was utilized. The two cell types were separated by using the transwell insert, which prevented direct physical contact between DRG neurons and DCs but allowed for mediators exchange. No difference of % pruritogen-responsive neurons was observed in the indirect co-culture system compared to the DRG monoculture (Fig. 5B). In the direct co-culture without stimulation, there was also no difference between the two groups (Fig. 5C).

In addition, the responsive neurons to the agonist of each receptor were measured by calcium imaging to investigate the activation of itch-related receptors including TRPA1, TRPV1, H1R, H4R and TRPV4. DRG co-cultured with DCs showed a higher percentage of neurons responding to agonists of TRPA1 (AITC: DRG monoculture 0.00% vs. DRG-DCs co-culture 0.59%, *p* < 0.05), H1R (HTMT: DRG monoculture 0.44% vs. DRG-DCs co-culture 2.13%, *p* < 0.01), TRPV4 (GSK1016790A: DRG monoculture 2.51% vs. DRG-DCs co-culture 5.45%, *p* < 0.01) and TRPV1 (capsaicin: DRG monoculture 20.39% vs. DRG-DCs co-culture 24.41%, *p* < 0.01) (*N* = 3, Fig. 5D and E). To determine whether DCs in our co-culture system were in an activated state, the content of TNF-α and IL-1β in the supernatant of the co-cultures was measured. A higher content of both TNF-α and IL-1β were detected when DRG neurons were directly co-cultured with stimulated DCs compared to indirect co-culture or direct co-culture without stimulation (*N* = 3, *p* < 0.05, Fig. 5F and G). In contrast, no detectable levels of these cytokines were found in any DRG monocultures.

In addition to investigating the activation of itch receptors by calcium imaging, their expression levels were determined by RT-qPCR. We compared the expression of several itch-related genes including *H1R*, *H2R*, *H3R*, *II31RA*, Mrgpr member a3 (*MRGPRA3*), Mrgpr member c11 (*MRGPRC11*), Mrgpr member d (*MRGPRAD*), *OSMR*, protease-activated receptor-2 *(PAR2)*, *TRPA1*, *TRPV1* and *TRPV4* between DRG in co-culture with DCs and DRG monoculture (Figure A2 in the additional file 2). Only for the *TRPV4* gene, a significant 3.4-fold increase in the expression of DRG of the co-culture system was observed (*p* < 0.05, *N* = 3, Fig. 5H and Figure A1.2 in the additional file 1). The expression of TRPV4 in DRG was also verified through an immunofluorescence investigation using specific markers, such as MHCII and NeuN, to distinguish between DCs and DRG neurons. The percentage of TRPV4^+^NeuN^+^ cells was calculated in relation to the total NeuN^+^ cells. It was found that the percentage of TRPV4^+^NeuN^+^ cells was higher in DRG of the co-culture system (DRG monoculture 86.69 ± 4.81% vs. DRG-DCs co-culture 94.04 ± 2.86%, *N* = 3, *p* < 0.05, Fig. 5I). These findings confirmed an increase expression of TRPV4 in DRG in co-culture with DCs.

## Discussion

Previously, signs of peripheral sensitization were observed in the TDI-induced ACD mouse model. This was accompanied by an increase of activated DCs in DRG of TDI-sensitized mice [11]. Nevertheless, how DCs interact with sensory neurons in DRG has remained unclear. Studies have suggested that immune cells in close proximity to sensory nerve fibers within the skin may contribute to peripheral sensitization by releasing inflammatory mediators that can sensitize sensory neurons [13]. However, the mechanism of peripheral sensitization is not fully elucidated. The objective of this study is to examine the role of MHCII^+^ cells in DRG under ACD condition with a focus on itch peripheral sensitization directly in the DRG. The TDI mouse model for ACD and the co-culture model of BMDCs and DRG neurons was employed in this study.

After sensitization and repetitive challenge with TDI, the mice exhibited the typical signs of ACD, including a notable increase in itch response, consistent with findings from previous studies [11, 19, 20]. Our findings indicated the presence of edema and inflammation, as observed by increased ear swelling, thickening of both the epidermal and dermal layers of the ear skin, and an influx of eosinophils into the ear skin. In addition, the total serum IgE level was markedly elevated in the TDI-sensitized group, indicating that these mice were in allergic condition. Overall, we successfully induced ACD by TDI.

Peripheral sensitization refers to the phenomenon by which sensory nerve fibers become more responsive to stimuli and their thresholds are lowered. This process is mediated by various substances released by neurons, immune cells, keratinocytes, and fibroblasts. These substances include ATP, prostaglandins, leukotrienes, nerve growth factor, IL-6, IL-1β, TNF-α and histamine [21]. Peripheral sensitization does not only play a role in ACD but also in allergic settings like atopic dermatitis (AD). A meta-analysis reported that AD patients displayed itch sensitization. AD patients showed increased sensitivity to histamine-induced itch in the lesional skin compared to healthy control [10]. Consistent with previous studies, the TDI-sensitized mice exhibited peripheral sensitization as indicated by a higher percentage of neurons in the excised DRG responding to IL-31 compared to the control group [11]. This finding was corroborated with present results as an elevated expression of *IL31RA* in DRG of the TDI-sensitized group was found. IL-31, a pruritogenic cytokine, is primarily produced by T helper 2 cells (Th2 cells). An itch signal is transmitted by a heterodimeric receptor composed of IL31RA and OSMR; both are expressed in DRG neurons. It has been shown that IL-31 transgenic mice as well as mice treated with IL-31 exhibit AD-like skin lesions and scratching behaviors [22, 23]. A Phase II clinical trial was carried out to assess the efficacy of Nemolizumab, a humanized monoclonal antibody targeting IL-31 receptors, in AD patients. The findings from this study demonstrated that treatment with the anti-IL-31 receptor antibody resulted in an improvement in both clinical symptoms associated with AD and pruritus [24]. Apart from IL-31, DRG excised from TDI-sensitized mice in our study also exhibited an increased sensitivity to histamine and a non-histaminergic pruritogen. Furthermore, we observed the activation of TRPV1 and TRPA1 in these excised DRG. Both TRPV1 and TRPA1 are ion channels found in DRG neurons and play a role in signaling itch sensation in response to a variety of mediators such as IL-31 and histamine [20]. In an experimental mouse model of ACD induced by squaric acid dibutyl ester (SADBE), both TRPA1 and TRPV1 channels have been shown to be essential for the transmission of itch sensations [25]. Overall, there is an increase of sensitivity at the peripheral level, alterations in neuronal responsiveness to pruritogens and activity of itch-related receptors in DRG of TDI-sensitized mice.

The increase of MHCII^+^ cells in DRG was found in the TDI-sensitized group indicating neuroinflammation. The neuroinflammation is defined by the infiltration of leucocytes and enhanced production of inflammatory mediators in the peripheral nervous system (peripheral nerves and ganglia) and central nervous system (spinal cord and brain) [26]. This result was consistent with a study of an airway allergic model induced by house dust mite [14]. Allergic airway inflammation resulted in an elevated ratio of dendritic cells to neurons compared to the control group. This was determined by assessing MHCII expression, which served as a marker for identifying DCs. They also investigated if DCs proliferated in the ganglia or migrated from the outside into the ganglia. The 5-ethynyl-2’-deoxyuridine (EdU) proliferation study provided evidence that DCs migrated from an external source into the ganglia. However, for our setting it is still unknown whether these MHCII^+^ cells proliferated within DRG or migrated from the outside. This aspect requires further investigation. In a study conducted by Fukuyama et al. in 2017 [11], it was also observed that the presence of TDI-induced ACD resulted in an increase in the percentage of CD11c^+^TNFα^+^ cells in DRG. This finding suggested that ACD is associated with an elevated number of these specific immune cells present in DRG. MHCII expression is not limited to DC but can also be found in other immune cells such as B-cells and macrophages. Our immunofluorescence analysis revealed that these MHCII^+^ cells in DRG might not be B-cells and M2 macrophages as no cells in DRG expressed CD19 or arginase. On the other hand, some MHCII^+^ cells in DRG expressed CD68, which is a typical marker for macrophages. CD68 is not only expressed in macrophages but also other cells of the mononuclear phagocyte lineage including DCs [27]. Based on the results of previous studies and our findings mentioned above, DCs are likely the main cell type of these MHCII^+^ cells in DRG under ACD condition.

Since previous studies indicated the migration of DCs into the ganglia during allergic condition [11, 14], DC could serve as representative MHCII^+^ cells in DRG. Additionally, there may be an association between neuroimmune interaction and itch sensitization. Thus, we conducted the co-culture experiment with DRG neurons and BMDCs to investigate the role of MHCII^+^ cells in DRG under ACD conditions in the context of peripheral sensitization. The co-culture was stimulated with Th2 cytokines as ACD against isocyanates is a disease associated with a type 2 immune response [28]. The number of pruritogen-responsive neurons increased in DRG co-cultured directly with DCs, indicating peripheral sensitization. Interestingly this difference was lost in the indirect co-culture setting and normal condition without stimulation. Therefore, direct cell-cell contact, and allergic condition seem crucial for their interaction.

Also, the activation of itch receptors including H1R, TRPA1, TRPV1 and TRPV4 was found in DRG co-cultured with DCs. Consistent with the in vivo study, TRPV1 and TRPA1 were also activated under ACD condition and played a vital role in itch transmission pathway as mentioned before [20, 25]. H1R has been one of the most popular drug targets of the allergic diseases for a decade since the first H1 antihistamine drug was launched into the market in 1942 [29–31]. Also, H1R, a histamine receptor, is expressed in sensory neurons and critically contributes to both peripheral and central itch transmission pathways [6]. From our study, we observed that certain DRG neurons exhibit a response to H2R and H4R agonists; however, there was no difference between the two groups. Notably, there was a substantial percentage of responsive neurons to the H2R agonist. It was found that intradermal injection of H2R agonist did not cause scratching and H2R antagonist did not reduce histamine induced itch in mice [32]. However, H2 antagonist facilitated the restoration of the skin barrier in mice with dry skin, while H2 agonist hindered this repair under similar condition [33]. Additionally, a combination treatment using an H2 agonist and H4 antagonist decreased skin inflammation and scratching in a mouse model of psoriasis [34]. Overall, the full understanding of the role of H2R in allergic contact dermatitis remains unclear.

TNF-α and IL-1β levels were elevated in the supernatant of DRG-DCs co-culture with stimulation, demonstrating activation of DCs. TNF-α and IL-1β are not only classical activation markers and pro-inflammatory cytokines of DC but they are also associated with sensitization of sensory neurons [35]. TNF-α/TNFR1 signaling is required for acute and chronic itch via peripheral and central mechanisms [36]. IL-1β released from immune and inflamed cells directly activated nociceptor to induce pain hypersensitivity in rat [37]. Therefore, these pro-inflammatory cytokines secreted by activated DCs could potentially act as mediators of peripheral sensitization in DRG neurons.

Comparing direct and indirect co-culture, it was evident that the levels of TNF-α and IL-1β were higher in the direct co-culture setting. Additionally, the increase in the number of pruritogen-responsive neurons was only observed in DRG directly co-cultured with DCs in the calcium imaging study. These findings highlight the essential role of direct cell-cell contact for interaction between DCs and DRG neurons. A previous study demonstrated the significant role of direct cell-cell contact in mediating immune cell responses when investigating interactions between DRG neurons and mast cells under allergic conditions. It was revealed that DRG neurons amplified mast cell response to antigen stimulation and activation of FcεRI receptors only in the direct co-culture but not in the indirect co-culture, leading to increased degranulation and elevated IL-6 secretion from mast cells [38].

In addition to study the activation of itch receptors by calcium imaging, these receptors were also investigated by their expressions via RT-qPCR. An upregulation of TRPV4 in DRG co-cultured with DCs was observed while the expression of H1R, TRPA1 and TRPV1 were not different between two groups. Previous studies showed a correlation between TRPV4 and itch behavior in both SADBE and dinitrofluorobenzene induced allergic contact dermatitis. Hapten and histamine-induced itch were decreased in TRPV4 knockout mice [39, 40]. Thus, targeting TRPV4 may be an effective way for alleviating itch. On the other hand, certain receptors such as H1R, TRPA1, and TRPV1 were activated without upregulation in their levels of activation. These results are consistent with a previous study that also found increased activity of itch receptors but no change in their expression levels [41]. Therefore, examining only expression levels may not be sufficient to determine the function of itch receptors. Further investigation, such as studying their activity through methods like calcium imaging, may be necessary. Overall, the co-culture study indicates that activated DCs stimulate itch receptors and elevate the number of neurons responsive to pruritogens, potentially contributing to peripheral sensitization in chronic pruritus caused by haptens such as TDI. The direct contact between cells is essential for this interaction.

## Conclusion

Our findings indicate that there is an increase of MHCII^+^ cells and peripheral itch sensitization in DRG under TDI-induced ACD condition. It has been found that MHCII^+^ cells in DRG might contribute to the peripheral sensitization of itch by activating itch receptors, as shown through co-culture studies between DRG neurons and DCs. Further studies are required to identify the specific mediator(s) responsible for peripheral sensitization induced by activated DCs.

### Electronic supplementary material

Below is the link to the electronic supplementary material.

Supplementary Material 1

Supplementary Material 2

## Data Availability

The datasets used and analyzed during the current study are available from the corresponding author on reasonable request.

## Abbreviations

ACD: Allergic contact dermatitis
IL-4: Interleukin 4
IL-13: Interleukin 13
IL-31: Interleukin 31
IL-6: Interleukin 6
DRG: Dorsal root ganglia
GPCRs: G-protein-coupled receptors
H1R-H4R: Histamine receptors 1–4
Mrgpr: Mas-related G protein-coupled receptor
TRPV1: Transient receptor potential channel vanilloid 1
DCs: Dendritic cells
TDI: Tolylene-2,4-diisocyanate
MHCII: Major histocompatibility complex class II
IgE: Immunoglobulin E
TRPA1: Transient receptor potential cation channel subfamily A member 1
AITC: Allyl isothiocyanate
IL31RA: IL-31 receptor A
OSMR: Oncostatin M receptor
RT-qPCR: Reverse transcription-quantitative polymerase chain reaction
CD68: Cluster of Differentiation 68
CD19: Cluster of Differentiation 19
BMDCs: Bone marrow derived dendritic cells
TRPV4: Transient receptor potential channel vanilloid 4
HTMT: Histamine trifluromethyl toluidine
ELISA: Enzyme-linked Immunosorbent Assay
TNF-α: Tumor necrosis factor alpha
IL-1β: Interleukin 1 beta
MRGPRA3: Mas-related G protein-coupled receptor member A3
MRGPRC11: Mas-related G protein-coupled receptor member C11
MRGPRD: Mas-related G protein-coupled receptor member D
PAR2: Protease-activated receptor-2
AD: Atopic dermatitis
Th2 cell: T helper 2 cell
TNFR1: Tumor necrosis factor receptor 1
SADBE: Squaric acid dibutyl ester
H&E: Hematoxylin and eosin
GAPDH: Glyceraldehyde-3-phosphate dehydrogenase
RPL13A: Ribosomal protein L13a
GM-CSF: Granulocyte–macrophage colony-stimulating factor
LPS: Lipopolysaccharide
FBS: Fetal bovine serum
