# Supplementary material for: Dendritic cells under allergic condition enhance the activation of pruritogen-responsive neurons via inducing itch receptors in a co-culture study
Source: BMC Immunol. 2024 Feb 12;25:17. doi: 10.1186/s12865-024-00604-4 (PMC10863282; doi:10.1186/s12865-024-00604-4)
Supplement: Supplementary file 1 — Supplementary Material 1 [file 12865_2024_604_MOESM1_ESM.docx]

**Additional file 1: Confirmation of the accuracy of end-point RT-qPCR product using agarose gel electrophoresis.**

Figure A1.1 showed the end-point RT-qPCR products of *IL31RA*, *OSMR*, *GAPDH* and *RPL13A* mRNA that were validated using agarose gel electrophoresis. You can see the results of the expression in Figure 2C and 2D.

Figure A1.2 showed the end-point RT-qPCR products of *TRPV4*, *GAPDH* and *RPL13A* mRNA that were validated using agarose gel electrophoresis. You can see the results of the expression in Figure 5H.

| **A B** |
| --- |
| 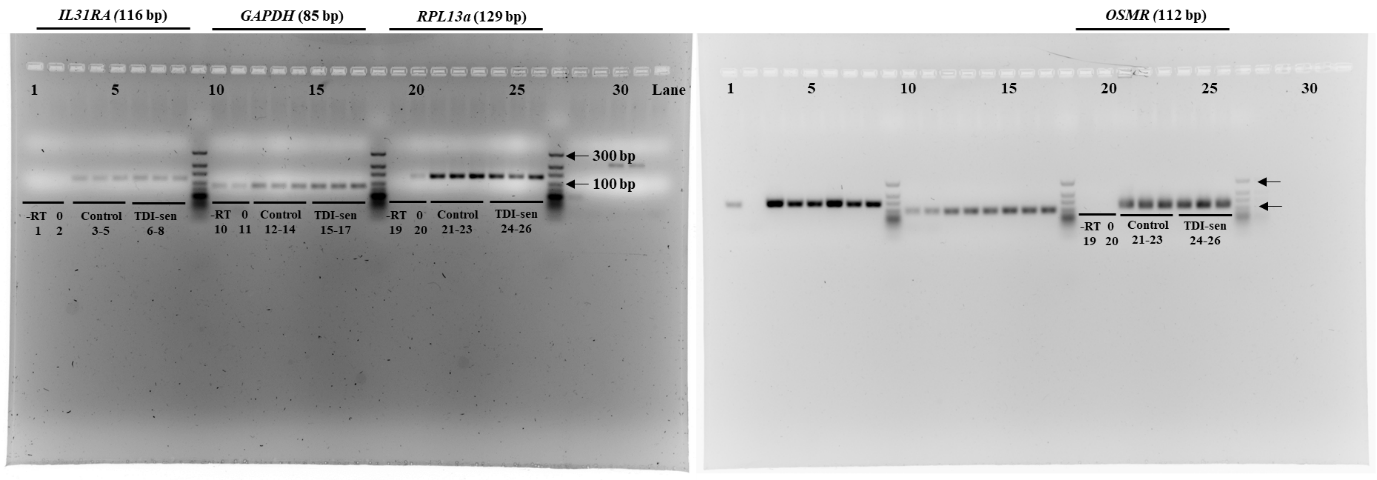 |
| **Figure A1.1** **Confirmation of the accuracy of end-point RT-qPCR product of *IL31RA* and *OSMR* gene using agarose gel electrophoresis.** -RT and 0 stand for minus reverse transcriptase control (MRT) and a no template control (NTC) respectively. There are three samples per group (N=3, control and TDI-sensitized group). |

| **A B** |
| --- |
| 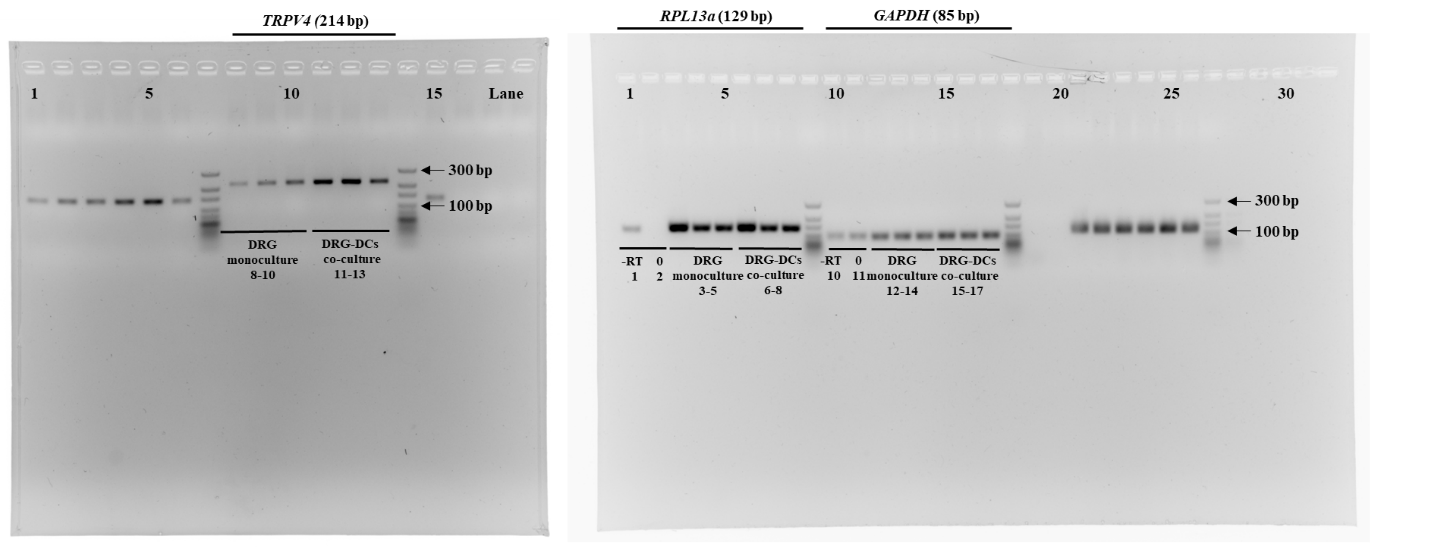 |
| **Figure A1.2 Confirmation of the accuracy of end-point RT-qPCR product of *TRPV4* gene using agarose gel electrophoresis.** -RT and 0 stand for minus reverse transcriptase control (MRT) and a no template control (NTC) respectively. There are three samples per group (N=3, control and TDI-sensitized group). |
