# Supplementary material for: Dendritic cells under allergic condition enhance the activation of pruritogen-responsive neurons via inducing itch receptors in a co-culture study
Source: BMC Immunol. 2024 Feb 12;25:17. doi: 10.1186/s12865-024-00604-4 (PMC10863282; doi:10.1186/s12865-024-00604-4)
Supplement: Supplementary file 2 — Supplementary Material 2 [file 12865_2024_604_MOESM2_ESM.docx]

**Additional file 2: The expression levels of itch receptor mRNA in DRG neurons co-cultured with BMDCs**

The expression levels of several itch receptor mRNA were determined by RT-qPCR. We compared the expression of several itch-related genes including *H1R, H2R, H3R TRPA1, II31RA, MRGPRA3, MRGPRC11, MRGPRD, OSMR, PAR2, TRPA1, TRPV1* and *TRPV4* between DRG neurons in co-culture with BMDCs and DRG monoculture (Figure A2.).

| 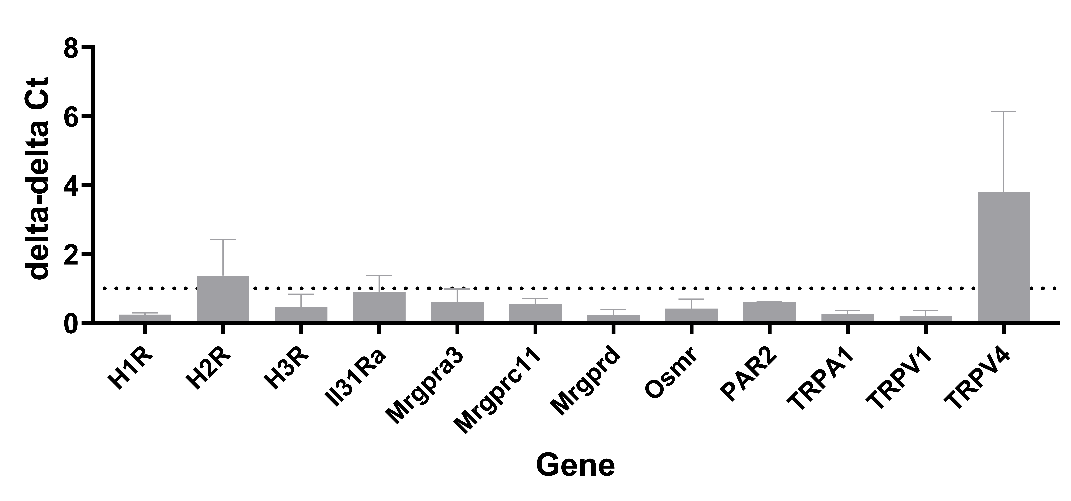 |
| --- |
| **Figure A2. The expression levels of itch receptor mRNA of DRG in co-culture with BMDCs compared to DRG monoculture.** Expression of itch-related genes including *H1R, H2R, H3R TRPA1, II31RA, MRGPRA3, MRGPRC11, MRGPRD, OSMR, PAR2, TRPA1, TRPV1* and *TRPV4* (ddCT/GAPDH and *RPL13A*) in DRG 24 h after direct co-culture with BMDCs (N=3, dotted line represents control level (DRG monoculture). |
